# Relations Between Bone Quantity, Microarchitecture, and Collagen Cross‐links on Mechanics Following In Vivo Irradiation in Mice

**DOI:** 10.1002/jbm4.10545

**Published:** 2021-09-26

**Authors:** Megan M Pendleton, Shannon R Emerzian, Saghi Sadoughi, Alfred Li, Jennifer W Liu, Simon Y Tang, Grace D O'Connell, Jean D Sibonga, Joshua S Alwood, Tony M Keaveny

**Affiliations:** ^1^ Department of Mechanical Engineering University of California Berkeley CA USA; ^2^ Endocrine Research Unit University of California and Veteran Affairs Medical Center San Francisco CA USA; ^3^ Department of Orthopaedic Surgery Washington University St. Louis MO USA; ^4^ Department of Biomedical Engineering Washington University St. Louis MO USA; ^5^ Department of Mechanical Engineering and Materials Science Washington University St. Louis MO USA; ^6^ Department of Orthopaedic Surgery University of California San Francisco CA USA; ^7^ Biomedical Research and Environmental Sciences Division NASA Johnson Space Center Houston TX USA; ^8^ Space Biosciences Division NASA Ames Research Center Moffett Field CA USA; ^9^ Department of Bioengineering University of California Berkeley CA USA

**Keywords:** AGING, BONE MECHANICS, FATIGUE, IONIZING RADIATION, RADIOTHERAPY, SPACEFLIGHT

## Abstract

Humans are exposed to ionizing radiation via spaceflight or cancer radiotherapy, and exposure from radiotherapy is known to increase risk of skeletal fractures. Although irradiation can reduce trabecular bone mass, alter trabecular microarchitecture, and increase collagen cross‐linking, the relative contributions of these effects to any loss of mechanical integrity remain unclear. To provide insight, while addressing both the monotonic strength and cyclic‐loading fatigue life, we conducted total‐body, acute, gamma‐irradiation experiments on skeletally mature (17‐week‐old) C57BL/6J male mice (*n* = 84). Mice were administered doses of either 0 Gy (sham), 1 Gy (motivated by cumulative exposures from a Mars mission), or 5 Gy (motivated by clinical therapy regimens) with retrieval of the lumbar vertebrae at either a short‐term (11‐day) or long‐term (12‐week) time point after exposure. Micro‐computed tomography was used to assess trabecular and cortical quantity and architecture, biochemical composition assays were used to assess collagen quality, and mechanical testing was performed to evaluate vertebral compressive strength and fatigue life. At 11 days post‐exposure, 5 Gy irradiation significantly reduced trabecular mass (*p* < 0.001), altered microarchitecture (eg, connectivity density *p* < 0.001), and increased collagen cross‐links (*p* < 0.001). Despite these changes, vertebral strength (*p* = 0.745) and fatigue life (*p* = 0.332) remained unaltered. At 12 weeks after 5 Gy exposure, the trends in trabecular bone persisted; in addition, regardless of irradiation, cortical thickness (*p* < 0.01) and fatigue life (*p* < 0.01) decreased. These results demonstrate that the highly significant effects of 5 Gy total‐body irradiation on the trabecular bone morphology and collagen cross‐links did not translate into detectable effects on vertebral mechanics. The only mechanical deficits observed were associated with aging. Together, these vertebral results suggest that for spaceflight, irradiation alone will likely not alter failure properties, and for radiotherapy, more investigations that include post‐exposure time as a positive control and testing of both failure modalities are needed to determine the cause of increased fracture risk. © 2021 The Authors. *JBMR Plus* published by Wiley Periodicals LLC on behalf of American Society for Bone and Mineral Research. This article has been contributed to by US Government employees and their work is in the public domain in the USA.

## Introduction

The effects of exposure to ionizing radiation on bone strength is of interest for numerous reasons, including oncologic radiotherapy and space exploration, among others. After oncologic radiotherapy, fragility fractures in cancer survivors are a known complication.^(^ ^1^, ^2^, ^3^, ^4^, ^5^, ^6^, ^7^, ^8^, ^9^, ^10^ ^)^ For example, the risk of hip insufficiency fracture in postmenopausal women treated for cervical, rectal, or anal cancer^(^ ^9^ ^)^ or in men treated for prostate cancer^(^ ^10^ ^)^ is increased at sites directly exposed to radiation (relative risk 1.66, 1.65, 3.14, and 1.76, respectively). Furthermore, a meta‐analysis reported a 14% incidence of pelvic insufficiency fractures after radiotherapy in patients with cervical cancer,^(^ ^1^ ^)^ noting fracture as a more common complication than previously thought, even with advances in treatment procedures that aim to minimize radiation to surrounding tissue, such as intensity‐modulated radiotherapy.^(^ ^2^, ^5^ ^)^ Thus, there is also a concern for bone degradation and fracture in astronauts, as they will be exposed to space radiation from solar and cosmic sources during deep‐space exploration.^(^ ^11^, ^12^, ^13^, ^14^, ^15^ ^)^

To address these complications and concerns, prior studies developed a mouse model to study radiation‐induced bone degradation.^(^ ^11^ ^)^ Although a significant body of literature has addressed irradiation and bone strength, using this model, a number of important issues remain unclear. First, because the effects of radiation on bone mechanics have primarily been studied for monotonic (one‐time) loading,^16^, ^17^, ^18^, ^19^, ^20^, ^21^, ^22^, ^23^ ^)^ much less is known about fatigue strength (cyclic loading). Because fractures after radiotherapy are commonly characterized as insufficiency fractures,^(^ ^24^, ^25^, ^26^, ^27^ ^)^ a type of fatigue fracture, a complete assessment of bone mechanical performance ought to include both monotonic and cyclic loading responses.^(^ ^28^ ^)^ Second, we have not identified the factor(s) responsible for elevated fracture risk. And third, in living bone, the effects of isolated doses of irradiation may evolve over time^(^ ^19^ ^)^ due to radiation effects on cellular activity.^(^ ^29^, ^30^ ^)^ Human clinical studies, to date, have demonstrated mixed results on cortical thinning and/or loss of bone mineral density after radiation therapy at various anatomical locations^(^ ^31^, ^32^, ^33^, ^34^ ^)^ and have not shown a conclusive relationship between bone loss and fracture incidence.^(^ ^2^, ^35^, ^36^ ^)^ Thus, mouse models have been critical for exploring the effect of radiation on bone quality^(^ ^37^ ^)^ and have addressed the effects of irradiation (total body or localized) on such morphological properties as bone mass, trabecular microarchitecture, and collagen structure.^(^ ^16^, ^17^, ^18^, ^19^, ^20^, ^21^, ^22^, ^23^, ^29^ ^)^ However, it still remains unclear which parameters are responsible for reduced skeletal integrity and how these relationships might change over time.

To provide insight into these issues, the main goal of this animal study was to investigate the effects in living mouse bone of different magnitudes of radiation exposure at different post‐exposure time points. To better understand the underlying biomechanics, we also sought to relate radiation‐induced changes in bone morphology at multiple scales (ie, whole‐bone to molecular level) with changes in mechanical properties. In particular, we addressed the effects of spacelike (1 Gy) and lower thresholds of clinically related (5 Gy) total‐body irradiation, and our assays included measurements of bone mass, bone microarchitecture (both cortical and trabecular), collagen structure, and both monotonic and cyclic mechanical properties.

## Materials and Methods

### Animals and experiment design

We conducted gamma‐irradiation experiments on mice at space‐ and clinically‐relevant doses and evaluated temporal effects by collecting bone tissue at both an early and late time point post‐irradiation. Assays included quantitative characterization of the trabecular and cortical bone mass and microarchitecture, biochemical assessments of the organic matrix, and mechanical characterization with monotonic and cyclic loading. Eighty‐four male C57BL/6J mice (Jackson Labs, Sacramento, CA, USA) were individually housed and randomly assigned to six groups (*n* = 14). At 17 weeks of age, the skeletally mature mice were exposed to either 0 Gy (sham‐irradiated), 1 Gy, or 5 Gy. Age, sex, and strain of mouse were chosen for mice to be near peak bone mass before radiation exposure and mice that exhibit skeletal changes typical of aging similar to that observed in humans.^(^ ^17^, ^38^, ^39^ ^)^ Mice were housed individually in standard cages with food (LabDiet 5001, Purina, St. Louis, MO, USA) and water *ad libitum*. Cages were located within a controlled animal facility room (24 ± 2°C, 55 ± 5% humidity, 12‐hour light/dark cycle). Mice were euthanized at two time points: either 11 days or 12 weeks post‐irradiation. Both are known time points to observe initial^(^ ^17^ ^)^ and lasting effects^(^ ^11^, ^22^, ^40^ ^)^ of radiation exposure. Investigators were blinded during animal allocation, animal handling, and endpoint measurements. All experiment procedures were conducted at NASA Ames Research Center and approved by the Institutional Animal Care and Use Committee (protocol #NAS‐13‐004‐Y3).

### ^137^Cs gamma irradiation

Conscious mice were exposed to total‐body, acute, γ‐radiation from a ^137^Cs source at 0.76 Gy min^−1^ (Mark‐3 Irradiator, J.L. Shepherd, San Fernando, CA, USA) for a dose of 1 and 5 Gy or were sham‐irradiated (0 Gy exposure), as reported elsewhere.^(^ ^17^ ^)^

### Specimen preparation

**Fig. 1:**
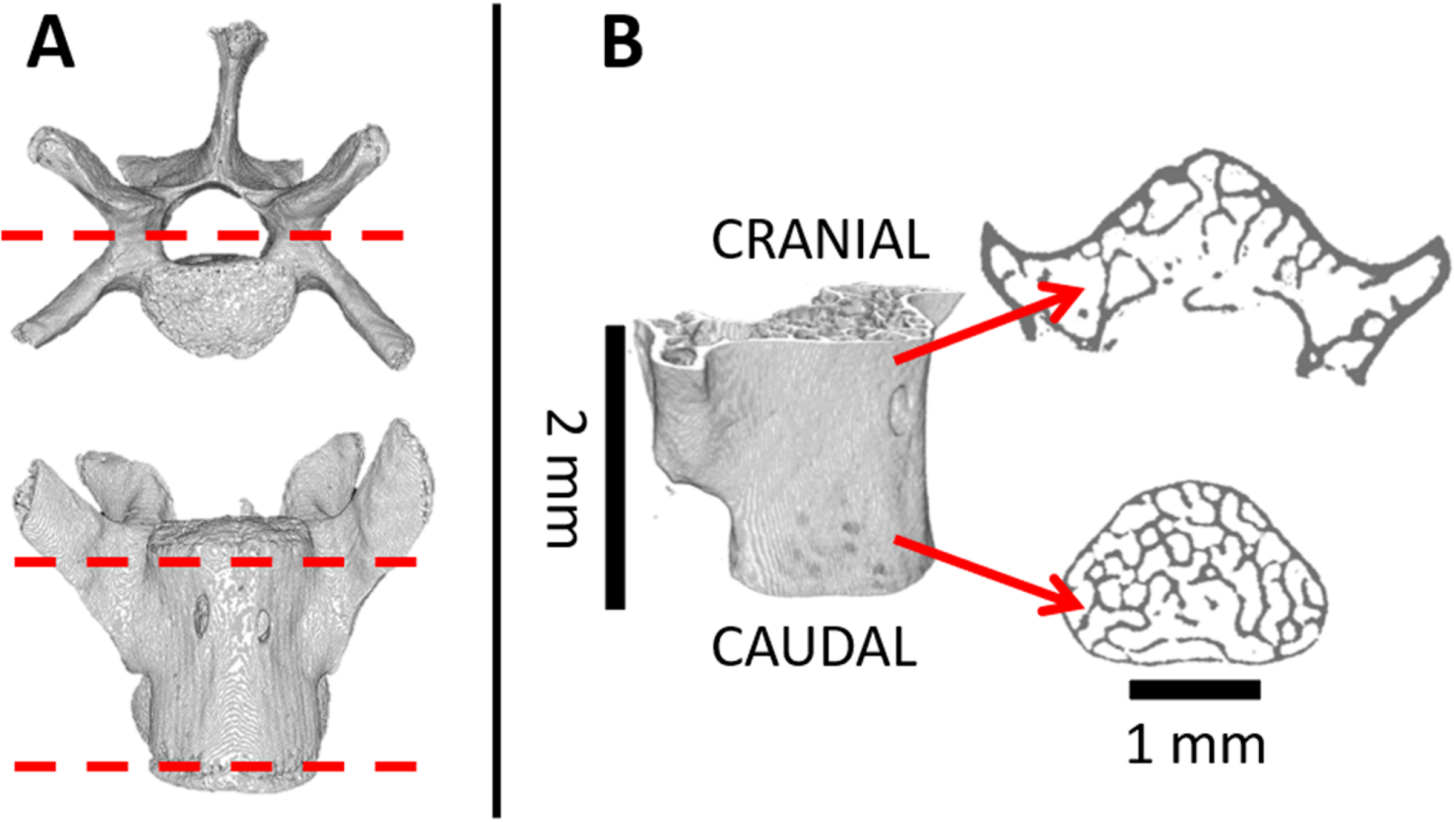
(*A*) L_5_ specimen before sample preparation. Red dotted lines indicate location of cuts applied using a diamond saw to remove posterior elements and achieve plano‐parallel surfaces for testing. (*B*) L_5_ specimen after sample preparation. Red arrows point to cross sections from respective cranial and caudal ends of vertebra.

After humane euthanization, lumbar and sacral vertebrae, L_3_ to S_1_, were excised from the mouse, gently cleaned of soft tissue, wrapped in saline‐soaked gauze (Gibco PBS, pH 7.4), and stored at −20°C. The endplates and posterior elements of the L_4_ and L_5_ were removed to produce plano‐parallel surfaces for uniaxial compression testing (Fig. 1). {FIG1} For further schematics on sample preparation, see Pendleton and colleagues.^(^ ^41^ ^)^ In total, there were three freeze–thaw cycles for L_4_ and L_5_ vertebrae (−20°C to room temperature) between dissection, specimen preparation, imaging, and mechanical testing.

### Quantitative micro‐CT imaging

After machining, the L_4_ and L_5_ specimens were imaged with quantitative micro‐CT (μCT 50, Scanco Medical AG, Bruttisellen, Switzerland) using a 10‐μm voxel size (55 kV, 109 μA, 1000 projections per 180°, 500 ms integration time). Micro‐CT images of the L_4_ and L_5_ specimens were analyzed for height (ImageJ 1.51h, Java 1.6.0). Additionally, the L_5_ specimens were evaluated for three‐dimensional architecture of the trabecular and cortical compartments (Scanco Evaluation Software v6.0). After manually segmenting the trabecular compartment—and using a lower threshold of 300 grayscale units and a Gaussian filter with sigma of 0.5 and support of 2—the following parameters were measured: trabecular bone volume fraction (BV/TV), number (Tb.N), thickness (Tb.Th), separation (Tb.Sp), connectivity density (Tb.Conn.D), structural model index (SMI), and volumetric bone mineral density (Tb.BMD). Additionally, after segmenting the cortex—from 0.5 mm above to 0.5 mm below the transverse processes and using a lower threshold of 380 grayscale units and a Gaussian filter with sigma of 0.8 and support of 1—the following parameters were measured: cortical thickness (Ct.Th), cross‐sectional area (CSA), and cortical volumetric bone mineral density (Ct.BMD). Final data for all micro‐CT analyses consisted of 13 to 14 samples in each group.

### Biochemical composition assays

After irradiation, two biochemical composition tests were conducted to assess the two primary molecular modifications thought to alter bone collagen after irradiation: (i) the accumulation of non‐enzymatic cross‐links and (ii) the fragmentation of the collagen backbone. Final data for all biochemical analyses consisted of 4 to 8 samples in each group for cross‐links and 4 to 5 samples in each group for fragmentation. The quantification of non‐enzymatic collagen cross‐links in the S_1_ vertebrae was achieved via a fluorometric assay that determined the relative fluorescence due to advanced glycation end products (AGEs)^(^ ^42^, ^43^, ^44^, ^45^ ^)^ relative to the amount of collagen in the bone matrix (protocol adapted from Sell and colleagues^(^ ^46^ ^)^). In brief, each S_1_ specimen was demineralized in 0.5 M ethylenediaminetetraacetic acid (EDTA) and hydrolyzed in 12 N HCl at 120°C for 3 hours to break down peptide bonds. The hydrolysate was then resuspended in PBS (0.1×) and pipetted in triplicate onto a black‐walled 96‐well plate. The non‐enzymatic collagen cross‐link content, or number of AGEs, was determined using fluorescence readings taken using a microplate reader (370 nm excitation, 440 nm emission). Readings were standardized to a quinine‐sulfate standard (quinine dissolved in H_2_SO_4_), and then normalized to the amount of collagen present in each sample, approximated by the amount of hydroxyproline.^(^ ^47^, ^48^, ^49^ ^)^

To measure collagen fragmentation, we used an automated electrophoresis assay (2100 Bioanalyzer, Agilent Technologies, Santa Clara, CA, USA) to assess the molecular weight distribution of collagen isolated from the L_3_ vertebrae, as described in detail elsewhere.^(^ ^49^ ^)^ Briefly, we first isolated the collagen from the L_3_ vertebrae via methods adapted from Burton and colleagues.^(^ ^50^ ^)^ The isolated collagen was then dissolved in 1× PBS, mixed with additional reagents (see Agilent Technologies Protein 230 Manual), and loaded on a bioanalyzer chip for automated electrophoresis. Rat‐tail collagen (C7661‐25MG; Sigma‐Aldrich, St. Louis, MO, USA) was run as a standard. The distribution of molecular weights of the bone collagen protein was assessed in two ways: (i) visually with a software‐generated “gel” and (ii) quantitatively with an electropherogram, a software‐generated fluorescence unit (FU) chart (Agilent 2100 Expert software). The nominal size of type I collagen, either alpha‐1 or alpha‐2, is between 130 and 150 kDa. To identify chain fragmentation, we looked for evidence of less protein in this range and a wider distribution of molecular weights. On the gel, this is observed as a lighter‐colored band or smeared band at ~150 kDa. On the electropherogram, fragmentation can be observed when the peak at ~150 kDa is diminished, indicating fewer fluorescence units and therefore fewer collagen chains of the nominal size. The quantification of collagen fragmentation was achieved via the software‐generated electropherogram by comparing the quantity of FU at the nominal collagen chain length (~150 kDa) for each group.

### Mechanical testing

After micro‐CT imaging, uniaxial compressive monotonic and cyclic mechanical tests were performed (TA ElectroForce 3200, Eden Prairie, MN, USA). Final data for all mechanical analyses consisted of 13 to 14 samples in each group for fatigue loading and 10 to 13 samples in each group for monotonic loading (excluding samples used to calibrate machine PID controller). Monotonic testing was conducted on the L_4_ specimens, at a platen displacement rate of 0.01 mm s^−1^. Force‐displacement data were collected (1000 Hz) and custom code (Matlab R2017a) was used to obtain whole‐vertebral stiffness (K), ultimate force (strength; F_ult_), and ultimate strain (ε_ult_). Cyclic testing was conducted on the L_5_ specimens, using methods described in detail elsewhere.^(^ ^41^ ^)^ Briefly, in order to load all specimens to the same initial elastic apparent strain, micro‐CT‐based finite element models were used to calculate specimen‐specific stiffnesses (K_FEA_) and minimum and maximum cyclic compressive forces, F_min_ and F_max_.^(^ ^41^ ^)^ After F_min_ and F_max_ values were calculated, specimens were cyclically loaded between F_min_ and F_max_ in uniaxial compression, with a sinusoidal waveform at 8 Hz until failure (TA ElectroForce 3200). Although faster than physiologic loading, 8 Hz was chosen to optimize test time while remaining below a frequency threshold of 15 Hz, known to alter fatigue behavior.^(^ ^51^, ^52^ ^)^ Testing was conducted at room temperature in a saline‐water bath to maintain hydration for the entire duration of the test. Specimen were placed, caudal‐side down, in the center of the fixed, lower platen. A pre‐load of 1 N was applied to obtain a flush, parallel mate between the specimen cranial surface and mobile, spherically seated upper platen. Cyclic testing began after the upper platen position was locked in place. Force‐displacement data were collected (1000 Hz), and custom code was used to obtain fatigue properties, including fatigue life (number of cycles to failure, N_f_), strain to failure (ε_f_), and specimen elastic stiffness (K_elastic_). Elastic modulus and ultimate stress were not calculated from experimental values because the cross‐sectional area of the vertebral body varies substantially from cranial to caudal ends, as is consistent with other rodent vertebra mechanical assessments.^(^ ^53^ ^)^ However, an average effective tissue modulus (E_tissue_) per vertebral specimen was calculated by comparing the ratio of computational to experimental whole‐bone stiffness (K_FEA_/K_elastic_) to the ratio of computational to experimental tissue modulus (10 GPa/E_tissue_).

### Statistics

For each of the 17 measurements (10× micro‐CT, 2× biochemical, 5× mechanical), an ANOVA was used to test for any significant effects of radiation dose (0, 1, or 5 Gy) and post‐exposure time (either 11 days or 12 weeks), including their interaction, in which both factors were treated as ordinal categorical variables (JMP Pro 15.0.0, SAS Institute Inc., Cary, NC, USA). The exact sample numbers used in each group for each experimental measurement are provided in Supplemental Table S1. For any parameter with a significant main effect of radiation dose, we assessed pairwise comparisons via Dunnett's post hoc test compared with 0 Gy. If the interaction factor was significant (*p* < 0.05), we used Dunnett's post hoc test to compare each group against a single baseline control (0 Gy, 11 days). Percent differences in measurements are reported in Table 1 {TBL 1}with respect to this baseline control, unless otherwise specified.

**Table 1:** Results of the ANOVA and Respective Dunnett's Post Hoc Tests Conducted for Parameters With Significance in the Main or Interaction Effect

Parameters	ANOVA			Baseline means and percent differences					
	Radiation dose	Post‐exposure time	Interaction	Control 0 Gy, 11 days	% Difference 1 Gy, 11 days	% Difference 5 Gy, 11 days	% Difference 0 Gy, 12 weeks	% Difference 1 Gy, 12 weeks	% Difference 5 Gy, 12 weeks
Microstructural									
BV/TV (%)	**0.000** ^a^	**0.001**	**0.022**	19.9 ± 2.6	−7.9	−22.3^***^	−11.5^**^	−8.1	−21.6^***^
Tb.Th (mm)	**0.000** ^a^	**0.024**	0.156	0.041 ± 0.001	−0.5	5.4	−3.2	0.0	4.8
Tb.Sp (mm)	**0.000** ^a^	**0.012**	0.272	0.195 ± 0.018	5.0	16.1	8.0	9.2	27.6
Tb.N (mm^−1^)	**0.000** ^a^	**0.000**	0.345	5.05 ± 0.43	−4.0	−11.8	−8.5	−9.1	−21.5
Conn.D (mm^−3^)	**0.000** ^a^	**0.000**	0.299	268 ± 35	−5.2	−17.0	−25.0	−29.7	−49.5
SMI	**0.000** ^a^	0.051	**0.000**	0.75 ± 0.21	20.1	107.5^***^	15.8	5.5	53.6^***^
Tb.BMD (mg of HAcm−^3^)	0.764	0.576	0.748	1070 ± 32	—	—	—	—	—
CSA (mm^2^)	0.379	0.866	0.883	0.64 ± 0.05	—	—	—	—	—
Ct.Th (mm)	0.489	**0.008**	0.924	0.054 ± 0.007	−4.0	0.5	−11.1	−13.0	−10.5
Ct.BMD (mg of HA cm^−3^)	0.946	0.837	0.848	1206 ± 33	—	—	—	—	—
Biochemical									
AGEs (ng quinine/mg collagen)	**0.000** ^a^	0.361	0.232	36.2 ± 3.4	−9.1	85.5	15.6	11.8	67.9
Fragmentation (FU)	0.265	0.770	0.400	213 ± 49	—	—	—	—	—
Mechanical									
Strength, F_ult_ (N)	0.745	0.193	0.564	32.5 ± 5.6	—	—	—	—	—
Ultimate Strain, ε_ult_ (%)	0.647	0.506	0.748	3.63 ± 0.37	—	—	—	—	—
Fatigue life log(N_f_)	0.332	**0.001**	0.453	5.14 ± 0.33	−3.2	−3.9	−10.0	−9.6	−15.1
Strain to Failure, ε_f_ (%)	0.716	0.408	0.244	3.18 ± 1.01	—	—	—	—	—
K_elastic_ (N mm^−1^)	0.447	**0.003**	0.077	1216 ± 105	‐4.6	−3.0	−11.2	−6.7	−16.5
K_FEA_ (N mm^−1^)	0.130	**0.000**	0.350	2180 ± 47.5	−2.6	−6.4	−13.1	−9.3	−15.6
E_tissue_ (GPa)	0.352	0.613	0.173	5.61 ± 0.15	—	—	—	—	—

BV/TV = bone volume fraction; Tb.Th = trabecular thickness; Tb.Sp = trabecular separation; Tb.N = trabecular number; Conn.D = connectivity density; SMI = structural model index; Tb.BMD = trabecular bone mineral density; CSA = cross‐sectional area; Ct.Th = cortical thickness; Ct.BMD = cortical bone mineral density; AGEs = advanced glycation end products; FU = fluorescence unit.

Control group data (0 Gy, 11 days) are shown as least squares means ± standard deviation. Percent differences compare respective group to a single control group (0 Gy, 11 days). ANOVA: Significant terms (*p* < 0.05) in **bold**. Respective Dunnett's test conducted for any parameter with significance in a main or interaction effect. For the main effects of Radiation dose:

^a^ p<0.05 for 5 Gy versus 0 Gy.

Parameters significant only for an interaction effect show:

^**^ *p* < 0.01 versus control.

^***^ *p* < 0.001 versus control.

To gain additional insight, we used correlation analysis and stepwise multiple regression analysis to identify structure–function relations between the underlying micro‐CT and biochemical variables with the mechanical properties (in particular, strength and fatigue life). First, a Pearson correlation analysis indicated which non‐mechanical variables were potentially significant (*p* < 0.10) with the mechanical properties (Pearson correlation coefficient, *r*). Next, we identified those variables that reached statistical significance (*p* < 0.05) in both the forward and backward multiple regression models; this analysis was repeated separately for monotonic strength and fatigue life as the outcome variable. For both analyses, all groups were pooled, and neither post‐exposure time nor radiation dose were accounted for.

## Results

### Effects on bone volume fraction (BV/TV)

BV/TV depended on radiation dose (*p* < 0.0001) and post‐exposure time (*p* < 0.001) and the interaction term (*p* < 0.05), indicating that the effects of radiation dose depended on post‐exposure time. Compared with the control group (0 Gy, 11 days), without irradiation BV/TV was lower by −11.5% at 12 weeks. For high‐dose irradiation (5 Gy), BV/TV was lower by −23% at 11 days (*p* < 0.0001) and by a similar amount at 12 weeks (−21.6%, *p* < 0.0001). For all variables affected by radiation dose (Table 1, *p* < 0.05 for “radiation dose”), the effects were consistently significant for 5 Gy but not significant for 1 Gy.

### Effects on other micro‐CT measurements

**Fig. 2:**
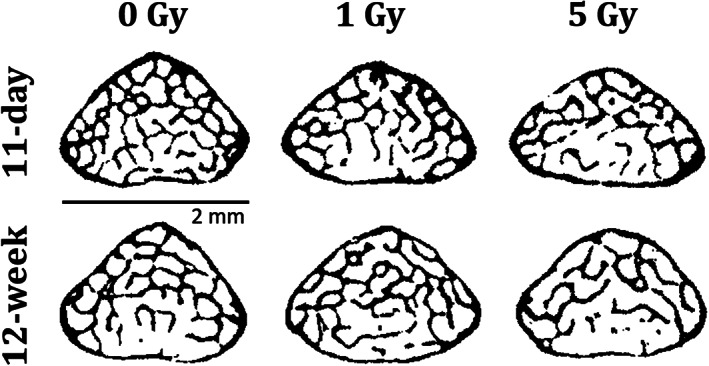
Micro‐CT images of transverse cross sections from the caudal region of representative L_5_ mouse vertebrae, showing the effects of three different one‐time radiation doses (0, 1, or 5 Gy) after 11 days or 12 weeks (all different animals).

**Fig. 3:**
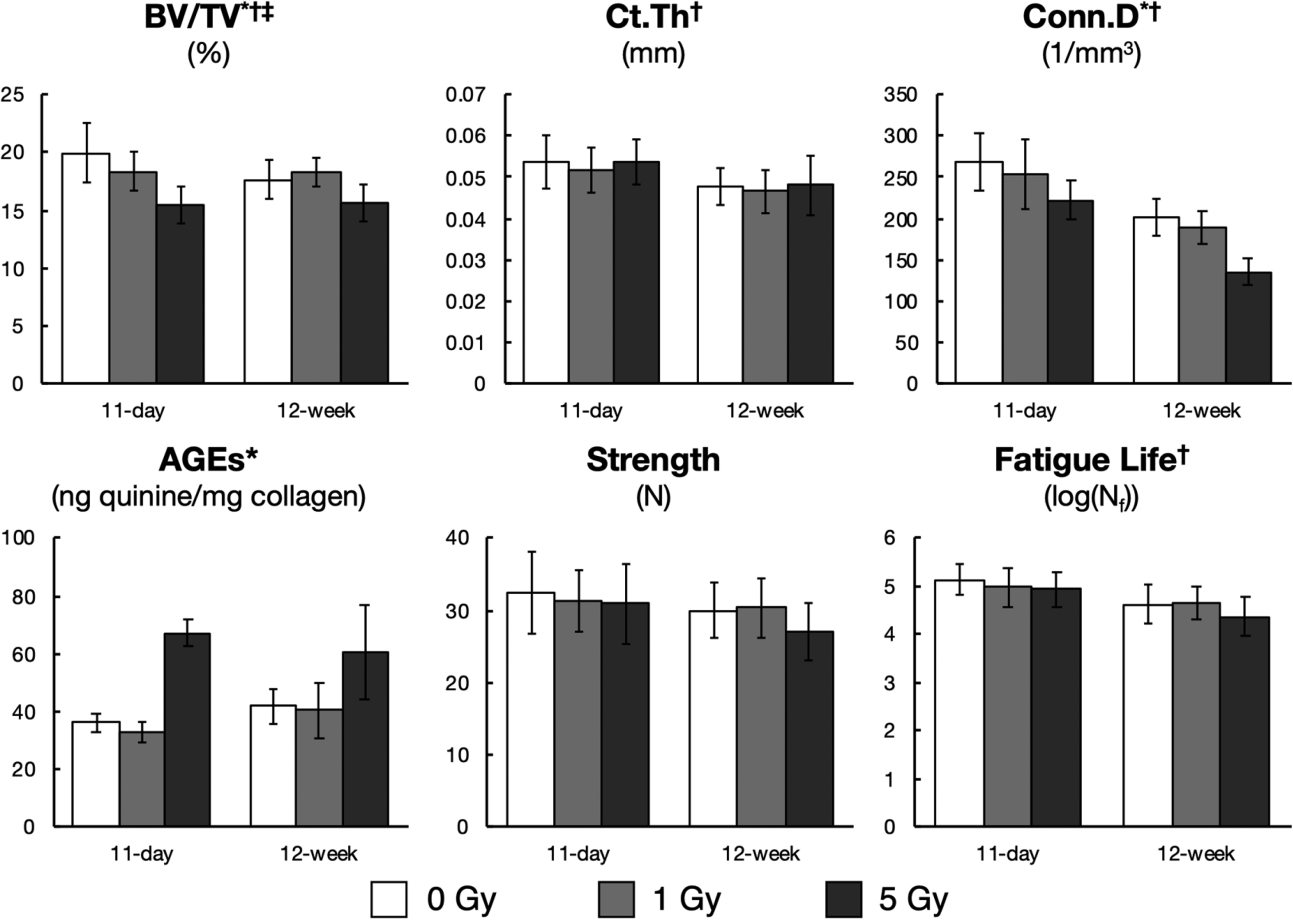
Effect of in vivo acute, total‐body gamma radiation (0, 1, and 5 Gy) at 11 days and 12 weeks post‐exposure on selected parameters. Data are shown as least‐square means; error bars represent standard deviation. Significance by ANOVA is indicated with superscripts: *radiation dose *p* < 0.05; ^†^post‐exposure time *p* < 0.05; or ^‡^interaction *p* < 0.05.

Most of the trabecular bone microarchitecture parameters were altered independently by radiation dose (*p* < 0.0001) and post‐exposure time (*p* < 0.05) (Table 1; images in Fig. 2). {FIG2} Compared with the control group, without irradiation trabecular Conn.D was lower by −25% at 12 weeks (*p* < 0.001) (Fig. 3). {FIG3} With high‐dose irradiation (5 Gy), Conn.D was lower by −17% (*p* < 0.001) within 11 days and by a greater amount after 12 weeks (−49.5%, *p* < 0.001). Similar trends were observed for Tb.N and Tb.Sp, with a lower number of trabeculae and greater spacing over time with irradiation (Table 1). Tb.Th was increased by 5 Gy irradiation, and the effect was the same at both 11 days (+5.4%, *p* < 0.01) and 12 weeks (+4.8%, *p* < 0.01). Changes in cortical bone parameters were not detected after irradiation. However, cortical thickness (Ct.Th) was associated with post‐exposure time (*p* < 0.01) and demonstrated cortical thinning in all the 12‐week groups (Table 1; Fig. 3).

### Effects on biochemical measurements of collagen

The only biochemical parameter significantly altered was the concentration of AGEs, which was only affected by radiation dose of 5 Gy (*p* < 0.0001). After 5 Gy irradiation, the number of AGEs was increased by +85.5% (*p* = 0.001) within 11 days and by a similar amount at 12 weeks (+67.9%, *p* = 0.002) (Table 1; Fig. 3). Changes were not detected in collagen fragmentation.

### Effects on mechanical properties

Of the five mechanical properties characterized, none were altered by radiation dose or a radiation dose by post‐exposure time interaction, while two parameters (fatigue life, K_elastic_) were altered by post‐exposure time (*p* < 0.01). At 12 weeks, fatigue life was lower by 10% (*p* = 0.002) without irradiation and lower by 9.6% (*p* = 0.004) and 15.1% (*p* < 0.001) with 1 and 5 Gy radiation exposure, respectively (Table 1; Fig. 3).

### Role of microarchitectural and biochemical measurements on mechanical properties

Results from the multiple regression analyses for all specimens indicated different structure–function relations between these measurements and the monotonic versus fatigue mechanical properties (Table 2). {TBL 2} Fatigue life was significantly associated with Ct.Th and Conn.D (adjusted *R* ^*2*^ = 0.29), although not significantly with any biochemical parameters. In contrast, monotonic strength was associated with biochemical AGEs (adjusted *R* ^*2*^ = 0.23).

**Table 2:** Independent Effect (Reported as Pearson's Correlation Coefficient) and Multivariate Regression Analysis of Microstructural and Biochemical Parameters on Measured Vertebral Strength (F_ult_) and Fatigue Life (log(N_f_))

Correlation analysis	F_ult_	log(N_f_)
Microstructural		
BV/TV	**+0.30** ^*^	+0.20^a^
Tb.Th	+0.02	−0.09
Tb.Sp	−**0.32** ^**^	−**0.37** ^***^
Tb.N	**+0.32** ^**^	**+0.40** ^***^
Conn.D	**+0.34** ^**^	**+0.49** ***
SMI	−0.16	−0.02
Tb.BMD	−0.09	−0.03
CSA	**+0.25** ^*^	+0.11
Ct.Th	+0.21^a^	**+0.35** ^**^
Ct.BMD	−0.11	−0.03
Biochemical		
AGEs	−**0.51** ^**^	−0.23
Fragmentation	−0.13	−0.24

Multivariate regression	F_ult_	log(N_f_)
Initial model^a^	BV/TV, Tb.N, Conn.D, CSA, Ct.Th, AGEs	BV/TV, Tb.N, Conn.D, Ct.Th
Final model		
Adjusted *R* ^*2*^	0.23	0.29
Parameter(s)	AGEs^**^	Conn.D^***^ Ct.Th^**^

BV/TV = bone volume fraction; Tb.Th = trabecular thickness; Tb.Sp = trabecular separation; Tb.N = trabecular number; Conn.D = connectivity density; SMI = structural model index; Tb.BMD = trabecular bone mineral density; CSA = cross‐sectional area; Ct.Th = cortical thickness; Ct.BMD = cortical bone mineral density; AGEs = advanced glycation end products.

Significant terms (*p* < 0.05) in **bold**. Tb.Sp and Tb.N were highly correlated; only Tb.N included in model.

^a^ Multivariate regression for parameters with *p* < 0.1 after correlation analysis.

^*^ *p* < 0.05.

^**^ *p* < 0.01.

^***^ *p* < 0.001.

## Discussion

Our results demonstrate that irradiation effects on bone morphology in mouse vertebra did not translate to detectable effects on overall bone mechanical properties, such as strength and fatigue life for 5 Gy total‐body irradiation. Specifically, 5 Gy irradiation caused acute deficits in trabecular mass and microarchitecture and increased collagen cross‐links but did not independently produce any measurable change in the compressive strength, fatigue life, or stiffness of the overall vertebral body. While consistent with previous reports showing irradiation acutely altering cancellous morphology (eg, rarefaction of the centrum's spongy cancellous tissue—loss of thin trabeculae and perforation of plates, leaving thicker, more widely spaced, more strutlike tissue^(^ ^54^ ^)^) driven by a temporal elevation of osteoclast activity^(^ ^17^, ^29^, ^30^, ^55^, ^56^ ^)^ and collagen cross‐link quantity,^(^ ^20^ ^)^ the lack of detectable mechanical effects in our experiment or computational models seems counterintuitive. One possible explanation is that a loss of strength from reduced trabecular BV/TV was offset by a gain in strength from increased collagen cross‐links. However, our univariate correlation analyses showed that radiation‐induced alterations to AGEs and microarchitecture parameters are both associated with a decrease in mechanical properties, ruling out this possibility. Another possibility is that irradiation may have altered the internal load sharing between the trabecular and cortical bone in such a way as to attenuate the biomechanical effects of any trabecular changes, a theory supported by prior finite element analyses of mouse vertebrae after irradiation^(^ ^18^ ^)^ and a topic of ongoing investigation. Regardless of the underlying mechanisms, our results nevertheless demonstrate that the highly significant observed effects of 5 Gy total‐body irradiation on the trabecular bone morphology and collagen cross‐linking did not translate into detectable effects on overall failure properties such as vertebral strength or fatigue life in this mouse vertebra model.

Two important features of our overall experimental design were (i) our inclusion of multiple assays that cover those usually only measured individually in other irradiation studies and (ii) our inclusion of post‐exposure time as a factor, enabling us to tease out time effects from the irradiation treatment effects. First, this approach enabled us to show that irradiation alone had no significant impact on mechanical failure properties in the short term; however, post‐exposure time, with and without irradiation did exhibit reductions in cyclic mechanics. We found an association between cortical thinning and reduced fatigue life that was only significant within all the 12‐week groups. It has been shown that the incidence of insufficiency fractures post‐radiotherapy increases after 1 year^(^ ^2^ ^)^ and that the median time to fracture after treatment is approximately 2.5 years.^(^ ^4^ ^)^ Taken together, these findings would question the role of irradiation alone and consider the role of irradiation plus time on insufficiency fractures,^24^, ^25^, ^26^, ^27^ ^)^ such as those after radiation therapy, which have been reported to occur after repetitive loading at magnitudes well below the bone's strength.^(^ ^57^, ^58^ ^)^ However, it is also possible that irradiation may affect adult human bone tissue differently than it affects mouse bone, a noteworthy limitation of our study, or our model has limitations as a clinical correlate, discussed further below. Interestingly, this effect of time had no impact on monotonic strength, underscoring an instance of greater sensitivity with cyclic testing to detect mechanical degradation in bone. Second, this approach enabled us to show 12 weeks of aging without irradiation‐diminished cancellous microarchitecture, cortical thickness, and fatigue life and, thus, served as a positive control in our study. Radiation exposure at 5 Gy altered these age‐related changes in key parameters, such that the acute irradiation effects on cancellous microarchitecture did not worsen with time (Table 1).

Our results confirm that although collagen cross‐links—also known as AGEs—are likely to have an effect on bone tissue material properties,^(^ ^47^ ^)^ AGEs alone cannot account for the irradiation‐induced degradation of bone fatigue life. Because an increase in collagen cross‐links has been widely observed in radiation experiments^(^ ^20^, ^59^, ^60^, ^61^, ^62^, ^63^, ^64^ ^)^ and associated with a reduction in monotonic bone strength in aging and diabetes,^(^ ^44^, ^48^, ^65^, ^66^, ^67^, ^68^, ^69^, ^70^, ^71^, ^72^, ^73^ ^)^ it has been hypothesized to cause diminished mechanical properties after irradiation.^(^ ^62^ ^)^ In the current study, 5 Gy irradiation doubled the concentration of fluorescent AGEs, consistent with previous studies.^(^ ^20^, ^59^, ^60^, ^61^, ^62^, ^63^, ^64^ ^)^ However, recent work suggests an increase in AGE content alone is not independently responsible for diminished mechanics.^(^ ^20^, ^49^ ^)^ In line with this, the twofold increase in AGEs observed in the current study did not coincide with reduced failure properties (5 Gy, 11 days; Table 1). This is further supported by our regression analysis, where AGEs were not an independent predictor of fatigue life but were the only predictor of monotonic strength.

Our study has a number of limitations. First, as noted above, a mouse model may not be a good model of human bone, which would be due to obvious differences in whole‐bone geometry and bone microstructure and also due to potential differences in tissue material behavior. Second, we focused our mechanical testing at the L_4_ and L_5_ vertebrae. Previous studies investigating the effects of irradiation and aging on trabecular bone loss have shown more pronounced bone loss in long bone (eg, tibial metaphysis) compared with the vertebral centrum in mouse^(^ ^17^, ^38^ ^)^ and human models.^(^ ^34^ ^)^ Thus, it is possible that mechanical testing at another skeletal site would have detected mechanical deficits. Third, there are limitations to this model from a spaceflight perspective. The space radiation environment is more complex than our gamma‐radiation model; in addition to low linear energy transfer (LET) gamma rays, spaceflight radiation also includes higher LET particles^(^ ^74^, ^75^ ^)^ such as protons, neutrons, and heavy ions, which will likely have greater impact on the bone than what we observed for 1 Gy gamma exposure.^(^ ^76^, ^77^, ^78^ ^)^ However, a spaceflight radiation dose will be received chronically over the course of a mission, not in a single acute dose as modeled in this study.^(^ ^79^ ^)^ Lastly, because this model used total‐body irradiation, there are certainly limitations when considering our results in the context of clinical applications. Conventional radiation therapy for tumors of the pelvis (eg, cervical, anal, rectal, prostate) is administered in a fractionated and localized manner (eg, 1.8 Gy for 30 days, for a total of 54 Gy), with surrounding bone tissue estimated to receive a dose up to 50% of the intended target area.^(^ ^12^ ^)^ Our model differs in that we administer a one‐time, much smaller dose of 5 Gy distributed across the whole body. Thus, it is possible that there are more significant systemic effects taking place in our model. It is also possible that our model is conservative with respect to clinical applications—with a dose larger than 5 Gy applied locally to the surrounding bone tissue, the bone quantity, quality, microarchitecture, and collagen structure may be further degraded^(^ ^20^ ^)^ such that they contribute to the increased risk in fracture.^(^ ^80^ ^)^

Despite these limitations, our results suggest implications with respect to future studies on spaceflight and clinical irradiation. For spaceflight applications, our results suggest that the expected radiation exposure per se on a roundtrip mission to Mars^(^ ^81^ ^)^ (ie, ~1 Sievert) will not significantly impact cyclic or monotonic mechanics. That said, in astronauts, the effects of microgravity result in an approximately 1% per month loss of spinal bone mineral density^(^ ^82^ ^)^ and both cortical and trabecular architecture^(^ ^83^ ^)^ due to lack of nominal mechanical loading of the bone. It is possible that any potential irradiation biomechanical effects might be accentuated in more porous bone. For spaceflight applications, future work that integrates unloading into our experimental protocol might therefore be informative. For clinical applications, extending our studies to more porous bones may have more relevance to humans because human bone typically has a lower bone volume fraction than the mouse bones used in this study. For both applications, one key finding from our study is that radiation treatment can alter major elements of centrum morphology, in mice, without having any appreciable effect on the bone's mechanical properties. Thus, quantification of centrum morphology is not a sufficient predictor of mechanics on its own in mice. As such, future in vivo studies investigating the effect of irradiation or the efficacy of suggested countermeasures (ie, antiresorptive drugs,^(^ ^55^, ^84^, ^85^, ^86^, ^87^, ^88^ ^)^ strength training,^(^ ^89^ ^)^ diet,^(^ ^90^, ^91^ ^)^ or some combination thereof^(^ ^92^ ^)^) require a detailed biomechanical assessment with architectural, mechanical, and biochemical quantification and inclusion of post‐exposure time as an added variable.

## Disclosures

TMK is a consultant for Amgen, AgNovos Healthcare, and O.N. Diagnostics and has equity in O.N. Diagnostics. All other authors state that they have no conflicts of interest.

## Supporting information


**Supplemental Table S1.** Number of Samples Collected for Each Experimental Group by Parameter

## References

[1] Chung YK , Lee YK , Yoon BH , Suh DH , Koo KH . Pelvic insufficiency fractures in cervical cancer after radiation therapy: a meta‐analysis and review. In Vivo. 2021;35(2):1109‐1115. 33622908 10.21873/invivo.12356 PMC8045108

[2] Salcedo MP , Sood AK , Jhingran A , et al. Pelvic fractures and changes in bone mineral density after radiotherapy for cervical, endometrial, and vaginal cancer: a prospective study of 239 women. Cancer. 2020;126(11):2607‐2613. 32125711 10.1002/cncr.32807 PMC7220839

[3] Sapienza LG , Salcedo MP , Ning MS , et al. Pelvic insufficiency fractures after external beam radiation therapy for gynecologic cancers: a meta‐analysis and meta‐regression of 3929 patients. Int J Radiat Oncol Biol Phys. 2020;106(3):475‐484. 31580930 10.1016/j.ijrobp.2019.09.012

[4] Kang YM , Chao TF , Wang TH , Hu YW . Increased risk of pelvic fracture after radiotherapy in rectal cancer survivors: a propensity matched study. Cancer Med. 2019;8(8):3639‐3647. 31104362 10.1002/cam4.2030 PMC6639197

[5] Bazire L , Xu H , Foy JP , et al. Pelvic insufficiency fracture (PIF) incidence in patients treated with intensity‐modulated radiation therapy (IMRT) for gynaecological or anal cancer: single‐institution experience and review of the literature. Br J Radiol. 2017;90(1073):20160885. 28291401 10.1259/bjr.20160885 PMC5605110

[6] Otani K , Teshima T , Ito Y , et al. Risk factors for vertebral compression fractures in preoperative chemoradiotherapy with gemcitabine for pancreatic cancer. Radiother Oncol. 2016;118(3):424‐429. 26806264 10.1016/j.radonc.2016.01.006

[7] Oh D , Huh SJ . Insufficiency fracture after radiation therapy. Radiat Oncol J. 2014;32(4):213‐220. 25568849 10.3857/roj.2014.32.4.213 PMC4282995

[8] Igdem S , Alco G , Ercan T , et al. Insufficiency fractures after pelvic radiotherapy in patients with prostate cancer. Int J Radiat Oncol Biol Phys. 2010;77(3):818‐823. 19879066 10.1016/j.ijrobp.2009.05.059

[9] Baxter NN , Habermann EB , Tepper JE , Durham SB , Virnig BA . Risk of pelvic fractures in older women following pelvic irradiation. JAMA. 2005;294(20):2587‐2593. 16304072 10.1001/jama.294.20.2587

[10] Elliott SP , Jarosek SL , Alanee SR , Konety BR , Dusenbery KE , Virnig BA . Three‐dimensional external beam radiotherapy for prostate cancer increases the risk of hip fracture. Cancer. 2011;117(19):4557‐4565. 21412999 10.1002/cncr.25994 PMC3135749

[11] Hamilton SA , Pecaut MJ , Gridley DS , et al. A murine model for bone loss from therapeutic and space‐relevant sources of radiation. J Appl Physiol (1985). 2006;101(3):789‐793. 16741258 10.1152/japplphysiol.01078.2005

[12] Willey JS , Lloyd SA , Nelson GA , Bateman TA . Space radiation and bone loss. Gravit Space Biol Bull. 2011;25(1):14‐21. 22826632 PMC3401484

[13] National Council on Radiation Protection and Measurements . Radiation protection guidance for activities in low‐earth orbit: recommendations of the National Council on Radiation Protection and Measurements. Bethesda, MD: National Council on Radiation Protection and Measurements. 2000;viii:210.

[14] Chancellor JC , Blue RS , Cengel KA , et al. Limitations in predicting the space radiation health risk for exploration astronauts. NPJ Microgravity. 2018;4(1):8. 29644336 10.1038/s41526-018-0043-2 PMC5882936

[15] Cucinotta FA , Durante M . Cancer risk from exposure to galactic cosmic rays: implications for space exploration by human beings. Lancet Oncol. 2006;7(5):431‐435. 16648048 10.1016/S1470-2045(06)70695-7

[16] Bandstra ER , Thompson RW , Nelson GA , et al. Musculoskeletal changes in mice from 20‐50 cGy of simulated galactic cosmic rays. Radiat Res. 2009;172(1):21‐29. 19580504 10.1667/RR1509.1

[17] Kondo H , Searby ND , Mojarrab R , et al. Total‐body irradiation of postpubertal mice with (137)Cs acutely compromises the microarchitecture of cancellous bone and increases osteoclasts. Radiat Res. 2009;171(3):283‐289. 19267555 10.1667/RR1463.1

[18] Alwood JS , Yumoto K , Mojarrab R , et al. Heavy ion irradiation and unloading effects on mouse lumbar vertebral microarchitecture, mechanical properties and tissue stresses. Bone. 2010;47(2):248‐255. 20466089 10.1016/j.bone.2010.05.004

[19] Wernle JD , Damron TA , Allen MJ , Mann KA . Local irradiation alters bone morphology and increases bone fragility in a mouse model. J Biomech. 2010;43(14):2738‐2746. 20655052 10.1016/j.jbiomech.2010.06.017

[20] Bartlow CM , Mann KA , Damron TA , Oest ME . Limited field radiation therapy results in decreased bone fracture toughness in a murine model. PLoS One. 2018;13(10):e0204928. 30281657 10.1371/journal.pone.0204928 PMC6169919

[21] Jia D , Gaddy D , Suva LJ , Corry PM . Rapid loss of bone mass and strength in mice after abdominal irradiation. Radiat Res. 2011;176(5):624‐635. 21859327 10.1667/rr2505.1 PMC3209530

[22] Oest ME , Policastro CG , Mann KA , Zimmerman ND , Damron TA . Longitudinal effects of single hindlimb radiation therapy on bone strength and morphology at local and contralateral sites. J Bone Miner Res. 2018;33(1):99‐112. 28902435 10.1002/jbmr.3289 PMC5776033

[23] Green DE , Adler BJ , Chan ME , et al. Altered composition of bone as triggered by irradiation facilitates the rapid erosion of the matrix by both cellular and physicochemical processes. PLoS One. 2013;8(5):e64952. 23741433 10.1371/journal.pone.0064952 PMC3669258

[24] Seong YJ , Shin JK , Park WR . Early detected femoral neck insufficiency fracture in a patient treated with long‐term bisphosphonate therapy for osteoporosis: a need for MRI. Int J Surg Case Rep. 2020;70:213‐215. 32417741 10.1016/j.ijscr.2020.04.003 PMC7229416

[25] Hatgis J , Granville M , Jacobson RE , Berti A . Sacral insufficiency fractures: recognition and treatment in patients with concurrent lumbar vertebral compression fractures. Cureus. 2017;9(2):e1008. 28293486 10.7759/cureus.1008 PMC5333948

[26] Tamaki Y , Nagamachi A , Inoue K , et al. Incidence and clinical features of sacral insufficiency fracture in the emergency department. Am J Emerg Med. 2017;35(9):1314‐1316. 28412162 10.1016/j.ajem.2017.03.037

[27] Griffith JF , Genant HK . New advances in imaging osteoporosis and its complications. Endocrine. 2012;42(1):39‐51. 22618377 10.1007/s12020-012-9691-2

[28] Hernandez CJ , van der Meulen MC . Understanding bone strength is not enough. J Bone Miner Res. 2017;32(6):1157‐1162. 28067411 10.1002/jbmr.3078 PMC5466476

[29] Turner RT , Iwaniec UT , Wong CP , et al. Acute exposure to high dose gamma‐radiation results in transient activation of bone lining cells. Bone. 2013;57(1):164‐173. 23954507 10.1016/j.bone.2013.08.002 PMC4042434

[30] Oest ME , Franken V , Kuchera T , Strauss J , Damron TA . Long‐term loss of osteoclasts and unopposed cortical mineral apposition following limited field irradiation. J Orthop Res. 2015;33(3):334‐342. 25408493 10.1002/jor.22761 PMC4382807

[31] Chen HH , Lee BF , Guo HR , Su WR , Chiu NT . Changes in bone mineral density of lumbar spine after pelvic radiotherapy. Radiother Oncol. 2002;62(2):239‐242. 11937252 10.1016/s0167-8140(02)00002-6

[32] Wei RL , Jung BC , Manzano W , et al. Bone mineral density loss in thoracic and lumbar vertebrae following radiation for abdominal cancers. Radiother Oncol. 2016;118(3):430‐436. 26993414 10.1016/j.radonc.2016.03.002

[33] Okoukoni C , Randolph DM , McTyre ER , et al. Early dose‐dependent cortical thinning of the femoral neck in anal cancer patients treated with pelvic radiation therapy. Bone. 2017;94:84‐89. 27780791 10.1016/j.bone.2016.10.021

[34] Hayar M , Durankus NK , Altun GD , Kocak Z , Uzal MC , Saynak M . Investigation of differences of sacral and vertebral bone mineral densities before and after radiotherapy in patients with locally advanced rectal cancer. Cancer Radiother. 2019;23(5):408‐415. 31331841 10.1016/j.canrad.2019.05.014

[35] Dhakal S , Chen J , McCance S , Rosier R , O'Keefe R , Constine LS . Bone density changes after radiation for extremity sarcomas: exploring the etiology of pathologic fractures. Int J Radiat Oncol Biol Phys. 2011;80(4):1158‐1163. 20888134 10.1016/j.ijrobp.2010.04.012

[36] Nambu A , Onishi H , Aoki S , et al. Rib fracture after stereotactic radiotherapy on follow‐up thin‐section computed tomography in 177 primary lung cancer patients. Radiat Oncol. 2011;6:137. 21995807 10.1186/1748-717X-6-137 PMC3213137

[37] Hernandez CJ , Keaveny TM . A biomechanical perspective on bone quality. Bone. 2006;39(6):1173‐1181. 16876493 10.1016/j.bone.2006.06.001 PMC1876764

[38] Glatt V , Canalis E , Stadmeyer L , Bouxsein ML . Age‐related changes in trabecular architecture differ in female and male C57BL/6J mice. J Bone Miner Res. 2007;22(8):1197‐1207. 17488199 10.1359/jbmr.070507

[39] Halloran BP , Ferguson VL , Simske SJ , Burghardt A , Venton LL , Majumdar S . Changes in bone structure and mass with advancing age in the male C57BL/6J mouse. J Bone Miner Res. 2002;17(6):1044‐1050. 12054159 10.1359/jbmr.2002.17.6.1044

[40] Bandstra ER , Pecaut MJ , Anderson ER , et al. Long‐term dose response of trabecular bone in mice to proton radiation. Radiat Res. 2008;169(6):607‐614. 18494551 10.1667/RR1310.1 PMC4416087

[41] Pendleton MM , Sadoughi S , Li A , O'Connell GD , Alwood JS , Keaveny TM . High‐precision method for cyclic loading of small‐animal vertebrae to assess bone quality. Bone Rep. 2018;9:165‐172. 30417036 10.1016/j.bonr.2018.10.002 PMC6222041

[42] Karim L , Bouxsein ML . Effect of type 2 diabetes‐related non‐enzymatic glycation on bone biomechanical properties. Bone. 2016;82:21‐27. 26211993 10.1016/j.bone.2015.07.028 PMC4679472

[43] Knott L , Bailey AJ . Collagen cross‐links in mineralizing tissues: a review of their chemistry, function, and clinical relevance. Bone. 1998;22(3):181‐187. 9514209 10.1016/s8756-3282(97)00279-2

[44] Burr DB . Changes in bone matrix properties with aging. Bone. 2019;120:85‐93. 30315999 10.1016/j.bone.2018.10.010

[45] Bailey AJ . Molecular mechanisms of ageing in connective tissues. Mech Ageing Dev. 2001;122(7):735‐755. 11322995 10.1016/s0047-6374(01)00225-1

[46] Sell DR , Monnier VM . Isolation, purification and partial characterization of novel fluorophores from aging human insoluble collagen‐rich tissue. Connect Tissue Res. 1989;19(1):77‐92. 2791558 10.3109/03008208909016816

[47] Barth HD , Zimmermann EA , Schaible E , Tang SY , Alliston T , Ritchie RO . Characterization of the effects of X‐ray irradiation on the hierarchical structure and mechanical properties of human cortical bone. Biomaterials. 2011;32(34):8892‐8904. 21885114 10.1016/j.biomaterials.2011.08.013 PMC4405888

[48] Tang SY , Zeenath U , Vashishth D . Effects of non‐enzymatic glycation on cancellous bone fragility. Bone. 2007;40(4):1144‐1151. 17257914 10.1016/j.bone.2006.12.056 PMC4398019

[49] Pendleton MM , Emerzian SR , Liu J , et al. Effects of ex vivo ionizing radiation on collagen structure and whole‐bone mechanical properties of mouse vertebrae. Bone. 2019;128:115043. 31445224 10.1016/j.bone.2019.115043 PMC6813909

[50] Burton B , Gaspar A , Josey D , Tupy J , Grynpas MD , Willett TL . Bone embrittlement and collagen modifications due to high‐dose gamma‐irradiation sterilization. Bone. 2014;61:71‐81. 24440514 10.1016/j.bone.2014.01.006

[51] Lafferty JF . Analytical model of the fatigue characteristics of bone. Aviat Space Environ Med. 1978;49(1 Pt. 2):170‐174. 623581

[52] Yamamoto E , Paul Crawford R , Chan DD , Keaveny TM . Development of residual strains in human vertebral trabecular bone after prolonged static and cyclic loading at low load levels. J Biomech. 2006;39(10):1812‐1818. 16038915 10.1016/j.jbiomech.2005.05.017

[53] Brouwers JE , Ruchelsman M , Rietbergen B , Bouxsein ML . Determination of rat vertebral bone compressive fatigue properties in untreated intact rats and zoledronic‐acid‐treated, ovariectomized rats. Osteoporos Int. 2009;20(8):1377‐1384. 19066708 10.1007/s00198-008-0803-z PMC2708332

[54] Alwood JS , Kumar A , Tran LH , Wang A , Limoli CL , Globus RK . Low‐dose, ionizing radiation and age‐related changes in skeletal microarchitecture. J Aging Res. 2012;2012:481983. 22570786 10.1155/2012/481983 PMC3337602

[55] Willey JS , Livingston EW , Robbins ME , et al. Risedronate prevents early radiation‐induced osteoporosis in mice at multiple skeletal locations. Bone. 2010;46(1):101‐111. 19747571 10.1016/j.bone.2009.09.002 PMC2818222

[56] Green DE , Adler BJ , Chan ME , Rubin CT . Devastation of adult stem cell pools by irradiation precedes collapse of trabecular bone quality and quantity. J Bone Miner Res. 2012;27(4):749‐759. 22190044 10.1002/jbmr.1505

[57] Kummari SR , Davis AJ , Vega LA , Ahn N , Cassinelli EH , Hernandez CJ . Trabecular microfracture precedes cortical shell failure in the rat caudal vertebra under cyclic overloading. Calcif Tissue Int. 2009;85(2):127‐133. 19488669 10.1007/s00223-009-9257-3

[58] Hernandez CJ , Lambers FM , Widjaja J , Chapa C , Rimnac CM . Quantitative relationships between microdamage and cancellous bone strength and stiffness. Bone. 2014;66:205‐213. 24928495 10.1016/j.bone.2014.05.023 PMC4125443

[59] Oest ME , Gong B , Esmonde‐White K , et al. Parathyroid hormone attenuates radiation‐induced increases in collagen crosslink ratio at periosteal surfaces of mouse tibia. Bone. 2016;86:91‐97. 26960578 10.1016/j.bone.2016.03.003 PMC4833661

[60] Gong B , Oest ME , Mann KA , Damron TA , Morris MD . Raman spectroscopy demonstrates prolonged alteration of bone chemical composition following extremity localized irradiation. Bone. 2013;57(1):252‐258. 23978492 10.1016/j.bone.2013.08.014 PMC3789379

[61] Mandair GS , Oest ME , Mann KA , Morris MD , Damron TA , Kohn DH . Radiation‐induced changes to bone composition extend beyond periosteal bone. Bone Rep. 2020;12:100262. 32258252 10.1016/j.bonr.2020.100262 PMC7125315

[62] Oest ME , Damron TA . Focal therapeutic irradiation induces an early transient increase in bone glycation. Radiat Res. 2014;181(4):439‐443. 24701964 10.1667/RR13451.1 PMC4382799

[63] Niehoff P , Wiltfang J , Springer IN , Weppner N , Kimmig B , Acil Y . Increased excretion of collagen crosslinks in irradiated patients indicates destruction of collagen. Int J Radiat Biol. 2006;82(7):503‐509. 16882622 10.1080/09553000600840948

[64] Chauhan S , Khan SA , Prasad A . Irradiation‐induced compositional effects on human bone after extracorporeal therapy for bone sarcoma. Calcif Tissue Int. 2018;103(2):175‐188. 29500623 10.1007/s00223-018-0408-2

[65] Saito M , Fujii K , Mori Y , Marumo K . Role of collagen enzymatic and glycation induced cross‐links as a determinant of bone quality in spontaneously diabetic WBN/Kob rats. Osteoporos Int. 2006;17(10):1514‐1523. 16770520 10.1007/s00198-006-0155-5

[66] Wang X , Shen X , Li X , Agrawal CM . Age‐related changes in the collagen network and toughness of bone. Bone. 2002;31(1):1‐7. 12110404 10.1016/s8756-3282(01)00697-4

[67] Vashishth D , Gibson GJ , Khoury JI , Schaffler MB , Kimura J , Fyhrie DP . Influence of nonenzymatic glycation on biomechanical properties of cortical bone. Bone. 2001;28(2):195‐201. 11182378 10.1016/s8756-3282(00)00434-8

[68] Poundarik AA , Wu PC , Evis Z , et al. A direct role of collagen glycation in bone fracture. J Mech Behav Biomed Mater. 2015;52:120‐130. 26530231 10.1016/j.jmbbm.2015.08.012 PMC4651854

[69] Paschalis EP , Shane E , Lyritis G , Skarantavos G , Mendelsohn R , Boskey AL . Bone fragility and collagen cross‐links. J Bone Miner Res. 2004;19(12):2000‐2004. 15537443 10.1359/JBMR.040820 PMC1456071

[70] Hernandez CJ , Tang SY , Baumbach BM , et al. Trabecular microfracture and the influence of pyridinium and non‐enzymatic glycation‐mediated collagen cross‐links. Bone. 2005;37(6):825‐832. 16140600 10.1016/j.bone.2005.07.019 PMC1876767

[71] Farlay D , Armas LA , Gineyts E , Akhter MP , Recker RR , Boivin G . Nonenzymatic glycation and degree of mineralization are higher in bone from fractured patients with type 1 diabetes mellitus. J Bone Miner Res. 2016;31(1):190‐195. 26234180 10.1002/jbmr.2607 PMC4876148

[72] Saito M , Marumo K . Collagen cross‐links as a determinant of bone quality: a possible explanation for bone fragility in aging, osteoporosis, and diabetes mellitus. Osteoporos Int. 2010;21(2):195‐214. 19760059 10.1007/s00198-009-1066-z

[73] Nyman JS , Roy A , Acuna RL , et al. Age‐related effect on the concentration of collagen crosslinks in human osteonal and interstitial bone tissue. Bone. 2006;39(6):1210‐1217. 16962838 10.1016/j.bone.2006.06.026 PMC1847577

[74] Chancellor JC , Scott GB , Sutton JP . Space radiation: the number one risk to astronaut health beyond low earth orbit. Life (Basel). 2014;4(3):491‐510. 25370382 10.3390/life4030491 PMC4206856

[75] Cucinotta FA , Cacao E . Non‐targeted effects models predict significantly higher Mars mission cancer risk than targeted effects models. Sci Rep. 2017;7(1):1832. 28500351 10.1038/s41598-017-02087-3 PMC5431989

[76] Yumoto K , Globus RK , Mojarrab R , et al. Short‐term effects of whole‐body exposure to (56)fe ions in combination with musculoskeletal disuse on bone cells. Radiat Res. 2010;173(4):494‐504. 20334522 10.1667/RR1754.1

[77] Alwood JS , Tran LH , Schreurs A‐S , et al. Dose‐and ion‐dependent effects in the oxidative stress response to space‐like radiation exposure in the skeletal system. Int J Mol Sci. 2017;18(10):2117. 10.3390/ijms18102117 PMC5666799 28994728

[78] Lloyd SA , Bandstra ER , Travis ND , et al. Spaceflight‐relevant types of ionizing radiation and cortical bone: potential LET effect? Adv Space Res. 2008;42(12):1889‐1897. 19122806 10.1016/j.asr.2008.08.006 PMC2603056

[79] Yu K , Doherty AH , Genik PC , et al. Mimicking the effects of spaceflight on bone: combined effects of disuse and chronic low‐dose rate radiation exposure on bone mass in mice. Life Sci Space Res (Amst). 2017;15:62‐68. 29198315 10.1016/j.lssr.2017.08.004

[80] Farris MK , Helis CA , Hughes RT , et al. Bench to bedside: animal models of radiation induced musculoskeletal toxicity. Cancers (Basel). 2020;12(2):427. 10.3390/cancers12020427 PMC7073177 32059447

[81] Hassler DM , Zeitlin C , Wimmer‐Schweingruber RF , et al. Mars' surface radiation environment measured with the Mars science laboratory's curiosity rover. Science. 2014;343(6169):1244797. 24324275 10.1126/science.1244797

[82] LeBlanc A , Schneider V , Shackelford L , et al. Bone mineral and lean tissue loss after long duration space flight. J Musculoskelet Neuronal Interact. 2000;1(2):157‐160. 15758512

[83] Vico L , van Rietbergen B , Vilayphiou N , et al. Cortical and trabecular bone microstructure did not recover at weight‐bearing skeletal sites and progressively deteriorated at non‐weight‐bearing sites during the year following International Space Station missions. J Bone Miner Res. 2017;32(10):2010‐2021. 28574653 10.1002/jbmr.3188

[84] Dhesy‐Thind S , Fletcher GG , Blanchette PS , et al. Use of adjuvant bisphosphonates and other bone‐modifying agents in breast cancer: a Cancer Care Ontario and American Society of Clinical Oncology clinical practice guideline. J Clin Oncol. 2017;35(18):2062‐2081. 28618241 10.1200/JCO.2016.70.7257

[85] Arrington SA , Fisher ER , Willick GE , Mann KA , Allen MJ . Anabolic and antiresorptive drugs improve trabecular microarchitecture and reduce fracture risk following radiation therapy. Calcif Tissue Int. 2010;87(3):263‐272. 20563797 10.1007/s00223-010-9390-z

[86] Arrington SA , Damron TA , Mann KA , Allen MJ . Concurrent administration of zoledronic acid and irradiation leads to improved bone density, biomechanical strength, and microarchitecture in a mouse model of tumor‐induced osteolysis. J Surg Oncol. 2008;97(3):284‐290. 18161868 10.1002/jso.20949

[87] Kohno N , Aogi K , Minami H , et al. Zoledronic acid significantly reduces skeletal complications compared with placebo in Japanese women with bone metastases from breast cancer: a randomized, placebo‐controlled trial. J Clin Oncol. 2005;23(15):3314‐3321. 15738536 10.1200/JCO.2005.05.116

[88] Tolia M , Zygogianni A , Kouvaris JR , et al. The key role of bisphosphonates in the supportive care of cancer patients. Anticancer Res. 2014;34(1):23‐37. 24403442

[89] Govey PM , Zhang Y , Donahue HJ . Mechanical loading attenuates radiation‐induced bone loss in bone marrow transplanted mice. PLoS One. 2016;11(12):e0167673. 27936104 10.1371/journal.pone.0167673 PMC5147933

[90] Schreurs A‐S , Shirazi‐Fard Y , Shahnazari M , et al. Dried plum diet protects from bone loss caused by ionizing radiation. Sci Rep. 2016;6:21343. 26867002 10.1038/srep21343 PMC4750446

[91] Arjmandi BH , Johnson SA , Pourafshar S , et al. Bone‐protective effects of dried plum in postmenopausal women: efficacy and possible mechanisms. Nutrients. 2017;9(5):496. 10.3390/nu9050496 PMC5452226 28505102

[92] Leblanc A , Matsumoto T , Jones J , et al. Bisphosphonates as a supplement to exercise to protect bone during long‐duration spaceflight. Osteoporos Int. 2013;24(7):2105‐2114. 23334732 10.1007/s00198-012-2243-z

