# Supplementary material for: Relations Between Bone Quantity, Microarchitecture, and Collagen Cross‐links on Mechanics Following In Vivo Irradiation in Mice
Source: JBMR Plus. 2021 Sep 26;5(11):e10545. doi: 10.1002/jbm4.10545 (PMC8567491; doi:10.1002/jbm4.10545)
Supplement: Supplementary file 1 — Supplemental Table S1. Number of Samples Collected for Each Experimental Group by Parameter [file JBM4-5-e10545-s001.docx]

**Supplemental Material**

Table 1: Number of samples collected for each experimental group by parameter.
